# 

**Published:** 1859-02

**Authors:** 


Vol. 1. FEBRUARY, 1859. No. 4.

THE

### DENTAL REPORTER:

AN INDEPENDENT JOURNAL,

DEVOTED TO

### Dental Progress, and Improvements

IN THE

**MANUFACTURE AND USE OF**

INSTRUMENTS AND MATERIALS.

EDITED BY JNO. T. TOLAND.

Published Quarterly by

JNO. T. TOLAND,

MANUFACTURER OF

DENTAL INSTRUMENTS AND WHOLESALE DEALER IN DENTISTS' MATERIALS.

38 West Fourth Street, Cincinnati.

*PRICE* 25 *CENTS A YEAR IN* ADVANCE, *SINGLE COPIES* 10 *CENTS.*

WRIGHTSON & Co., Printers, 167 Walnut St., Cin

